# Analysis of bovine blastocysts indicates ovarian stimulation does not induce chromosome errors, nor discordance between inner-cell mass and trophectoderm lineages

**DOI:** 10.1016/j.theriogenology.2020.11.021

**Published:** 2021-02

**Authors:** D.A.R. Tutt, G. Silvestri, M. Serrano-Albal, R.J. Simmons, W.Y. Kwong, G. Guven-Ates, C. Canedo-Ribeiro, R. Labrecque, P. Blondin, A.H. Handyside, D.K. Griffin, K.D. Sinclair

**Affiliations:** a School of Biosciences, University of Nottingham, Sutton Bonington, LE12 5RD, UK; b School of Biosciences, University of Kent, Canterbury, CT2 7NH, UK; c Paragon Veterinary Group, Townhead Road, Dalston, Carlisle, CA5 7JF, UK; d L’Alliance Boviteq Inc, Saint-Hyacinthe, Québec, Canada

**Keywords:** Aneuploidy, Cattle, Ovarian stimulation, *In vitro* culture, Blastocyst, Biopsy

## Abstract

Contemporary systems for oocyte retrieval and culture of both cattle and human embryos are suboptimal with respect to pregnancy outcomes following transfer. In humans, chromosome abnormalities are the leading cause of early pregnancy loss in assisted reproduction. Consequently, pre-implantation genetic testing for aneuploidy (PGT-A) is widespread and there is considerable interest in its application to identify suitable cattle IVP embryos for transfer. Here we report on the nature and extent of chromosomal abnormalities following transvaginal follicular aspiration (OPU) and IVP in cattle. Nine sexually mature Holstein heifers underwent nine sequential cycles of OPU-IVP (six non-stimulated and three stimulated cycles), generating 459 blastocysts from 783 oocytes. We adopted a SNP-array approach normally employed in genomic evaluations but reanalysed (Turner et al., 2019; *Theriogenology***125**: 249) to detect levels of meiotic aneuploidy. Specifically, we asked whether ovarian stimulation increased the level of aneuploidy in either trophectoderm (TE) or inner-cell mass (ICM) lineages of blastocysts generated from OPU-IVP cycles. The proportion of Day 8 blastocysts of inseminated was greater (P < 0.001) for stimulated than non-stimulated cycles (0.712 ± 0.0288 vs. 0.466 ± 0.0360), but the overall proportion aneuploidy was similar for both groups (0.241 ± 0.0231). Most abnormalities consisted of meiotic trisomies. Twenty *in vivo* derived blastocysts recovered from the same donors were all euploid, thus indicating that 24 h of maturation is primarily responsible for aneuploidy induction. Chromosomal errors in OPU-IVP blastocysts decreased (P < 0.001) proportionately as stage/grade improved (from 0.373 for expanded Grade 2 to 0.128 for hatching Grade 1 blastocysts). Importantly, there was a high degree of concordance in the incidence of aneuploidy between TE and ICM lineages. Proportionately, 0.94 were “perfectly concordant” (i.e. identical result in both); 0.01 were imperfectly concordant (differing abnormalities detected); 0.05 were discordant; of which 0.03 detected a potentially lethal TE abnormality (false positives), leaving only 0.02 false negatives. These data support the use of TE biopsies for PGT-A in embryos undergoing genomic evaluation in cattle breeding. Finally, we report chromosome-specific errors and a high degree of variability in the incidence of aneuploidy between donors, suggesting a genetic contribution that merits further investigation.

## Highlights

• *In vitro* oocyte maturation rather than ovarian stimulation contributes to aneuploidy.
• Trophectoderm biopsies accurately represent the ploidy status of the overall embryo.
• SNP-array analyses facilitate simultaneous genomic evaluation and aneuploidy screening.
• Incidence of aneuploidy declines in developmentally more advanced embryos.
• High degree of variability in the incidence of aneuploidy exists between donors.

## 1 Introduction

The development of optimal systems for oocyte retrieval and maturation is a priority for cattle IVP and human assisted reproduction in order to maximise pregnancy outcomes following embryo transfer (ET). Maturational processes in growing oocytes are key to the success of post-fertilization development as chromosome segregation errors may originate at this stage. Optimising these processes to ensure faithful chromosome segregation is, therefore, the primary rate-limiting step in the successful implementation of systems for *in vitro* culture (IVC) of mammalian embryos [[1], [2], [3]]. Considerable progress has been made in developing systems for *in vitro* maturation (IVM) of germinal-vesicle (GV) stage oocytes from non-stimulated ovarian cycles [4,5]. Deficiencies remain, however, that limit the widespread uptake (although not interest) of oocyte retrieval from such cycles in commercial bovine (*Bos taurus*) *in vitro* embryo production (IVP). As an alternative, approaches that entail controlled ovarian stimulation and retrieval, followed by a period of maturation *in vitro* [6,7] are favored.

Protocols have been developed [8] and refined [9] in cattle based on the concept that, within stimulated cycles, a short period of gonadotrophin withdrawal (‘coasting’) prior to follicular aspiration can enhance the acquisition of developmental competency by oocytes [10]. These protocols can lead to high yields of transferrable-quality blastocysts per donor cycle. However, molecular mechanisms operating within the follicular compartment that may be predictive of subsequent oocyte development from such cycles are only just being elucidated [11]. Moreover, there is limited published data on pregnancy outcomes following embryo transfer (ET) [12]. Microarray-based transcript analyses of oocytes from ‘coasting protocols’ of differing duration reveal that genes involved in DNA recombination, replication and repair, together with those involved in spindle integrity and chromosome stability, are highly labile [13]. In humans, the extent to which ovarian stimulation can induce chromosome abnormalities (most of which are lethal) is controversial [14], and it also remains to be determined if ovarian stimulation induces chromosomal abnormalities in cattle.

The trend in human ART over the past decade has been to move towards extended culture to the blastocyst stage, with emphasis placed on generating at least one karyotypically normal blastocyst, determined by preimplantation genetic testing for aneuploidy (PGT-A) following sampling by trophectoderm (TE) biopsy [15]. This shift in emphasis has been facilitated to a large extent by rapid improvements in diagnostic approaches such as array comparative genomic hybridization (aCGH) and next-generation sequencing (NGS) that allow comprehensive chromosome screening [15]. Approaches involving single-nucleotide polymorphism (SNP) arrays to detect monogenic and chromosomal disorders simultaneously in human pre-implantation embryos are also gaining in popularity [16,17]. We have recently applied this technology to cattle IVP and reported the first live births [18]. Detection by this approach reveals that around 34% of human and 31% of cattle blastocysts are aneuploid [19].

Whilst the study of chromosome abnormalities in cattle embryos and ongoing pregnancies is still in its infancy, they are established as the leading cause of pregnancy loss, IVF failure, placental insufficiency and outlier birth weights in humans [[20], [21], [22]]. In cattle, some breeding (AI) bulls are screened for balanced chromosome translocations because of deleterious effects that chromosome abnormalities can have on future pregnancies [23]. In humans, there is convincing evidence that PGT-A can reduce miscarriage rates and improve time to first pregnancy [15]. However, PGT-A has not been established in commercial bovine IVP-ET despite the fact reduction in pregnancy loss following ET is a key consideration. This is the subject of ongoing studies at our collaborative laboratories.

With the foregoing discussion in mind, the current study sought to establish the nature and incidence of chromosomal abnormalities in both stimulated (coasting) and non-stimulated cycles of transvaginal follicular aspiration (Ovum Pick-Up; OPU) and IVP in cattle. Specifically, we tested the hypothesis that ovarian stimulation significantly increases the yield of oocytes for insemination but not at the expense of an increase in chromosome abnormalities. In order to achieve this, we employed a SNP-array platform that was recently adapted for PGT-A in cattle [18]. This makes use of data generated from the GGP 50K SNP array (Neogen Europe Ltd, UK) used routinely to establish cattle genomic estimated breeding values (gEBVs). The attractiveness of this approach is that, from TE biopsies, it could present breeders with the opportunity to make selection decisions based both on the genetic merit (gEBV) of the embryo [24] and, following PGT-A, its ploidy status. Theoretically, therefore, its potential to give rise to a viable pregnancy following ET should be improved. A further objective of the current study was to test the hypothesis that there is complete (or near complete) concordance in terms of chromosomal abnormalities between the TE (from where the cells are sampled) and the inner-cell mass (ICM). Whilst high concordance has been observed for human blastocysts [25,26], this is yet to be established for cattle. Such information will be essential when considering implementation of a cattle PGT-A programme, as selection decisions about the genome of the ICM derived fetus are based solely on information gleaned from TE biopsies.

## 2 Materials and methods

### 2.1 Generic considerations

All procedures adhered to the Animals (Scientific Procedures) Act, 1986. Associated protocols complied with the ARRIVE guidelines and were approved by the University of Nottingham Animal Welfare and Ethical Review Body (AWERB). All chemicals and reagents were sourced through Sigma-Aldrich Company Ltd (Dorset, UK) unless otherwise specified.

### 2.2 Animals and transvaginal follicular aspiration

Nine sexually mature 16-20 month-old virgin Holstein-Friesian heifers underwent initial estrous synchronization. Briefly, an intravaginal progesterone (P4)-releasing device (CIDR Vaginal Delivery System, 1.38g, Zoetis UK Ltd, Leatherhead, UK) was inserted and a GnRH analogue (Acegon, Zoetis UK Ltd, Leatherhead, UK) administered (2 mL i. m.). The prostaglandin (PGF_2α_) analogue Cloprostenol (Estrumate, MSD Animal Health, UK) was administered (2 mL i. m.) six and seven days later, at which time the CIDR was removed. GnRH (2 mL i. m.) was administered again 48 h later in order to assist ovulation.

Transvaginal ovarian follicular aspiration (ovum pick-up; OPU) commenced 4 d after estrus onset and was repeated twice weekly (every 3–4 days) for 3 weeks, leading to a total of 6 cycles of ‘Non-stimulated’ OPU. All OPU procedures were undertaken in a dedicated theatre where the ambient temperature was maintained between 28 and 33 °C. Cumulus-oocytes complexes (COCs) were aspirated as described previously [18]. Briefly, OPU used a Cook Medical vacuum pump with a 7.5 MHz ultrasound scanner (Exapad, IMV Imaging, Glasgow, UK) with aspiration pressure set at 70 mmHg. COCs were aspirated through an 18G needle and 1.4 m of 1.4 mm (I.D.) silicone tubing into 5 mL of Tyrodes lactate (TL) based aspiration media, as described previously [9].

Stimulated cycles of OPU using the same donors followed immediately and were undertaken once every 14 days over a five-week period, leading to a total of 3 cycles. The protocol commenced with ablation (aspiration) of all follicles ≥ 5 mm in diameter (dominant follicle removal; DFR) five days after the final non-stimulated session of OPU. A CIDR was inserted following DFR and ovarian stimulation commenced 48 h later. This involved six injections (i.m.) of follicle stimulating hormone (FSH; Folltropin, 70IU dose per injection, Vetoquinol UK Ltd, Towcester, UK) given at 12 h intervals. The first stimulated session of OPU was undertaken approximately 38–42 h following final FSH injection. The procedure for OPU and oocyte collection was as described for non-stimulated cycles. Following OPU, a replacement CIDR was inserted and the subsequent cycle of DFR commenced 8 days later.

### 2.3 In vitro embryo production (IVP)

OPU aspirants were passed through a heated (∼37 °C) filter, and filtrates transferred to 100 mm petri dishes on a heated stage (∼38 °C) for COC retrieval. COCs were graded 1–4 according to Ref. [27,28]. All COCs with sparse, expanded or absent cumulus or with fragmented, pale or irregular cytoplasm were classed Grade 4 and rejected. Grade 1–3 COCs were matured as previously described [18] in HEPES buffered TCM199 media supplemented with 10% (v/v) FBS, 4 mg/mL fatty acid free BSA, 0.2 mM pyruvate, 50 μg/mL gentamicin, 5 μg/mL FSH, 0.5 μg/mL LH, 1 μg/mL E_2_, in a screw top cryovial (Thermo Fisher Scientific Inc. Loughborough, UK) at atmospheric CO_2_ and 38.5 °C, for 23–24 h.

Fertilization occurred in 50 μL drops (5 oocytes/drop) under oil. Media consisted of modified TL media as previously described [9] supplemented with 0.6% (w/v) fatty acid free BSA, 1.5 μg/mL heparin, 0.2 mM sodium pyruvate, 0.08 mM penicillamine, 0.04 mM hypotaurine, 10 mM epinephrine, 50 μg/mL gentamicin. Frozen/thawed semen from a single sire was used throughout. Sperm preparation was by centrifugation through a 45%/90% BoviPure (Nidacon, Mölndal, Sweden) gradient. 2 μL of sperm preparation was added to each drop to give a final concentration of 70,000 sperm per drop. Oocytes and sperm were co-incubated for 18–21 h in a humidified environment of 5% CO_2_ in air at 38.5 °C.

Embryos were cultured (up to 11 per 10 μL drop) in modified SOF based sequential culture media (mSOF), as previously described [9], in a humidified environment under oil at 5% CO_2_, 5% O_2_ and 38.5 °C. Cleavage was assessed on Day 2 (30 h following transfer) and embryos classified according to cell number (i.e. 1, 2–3, and >4 cells). Embryos were transferred approximately 42 h later (Day 4) to 10 μL drops of the second culture media. Progression to morula was assessed 48 h later (Day 6), and embryos transferred to 20 μL drops of the third culture media. Embryos were assessed again 48 h later (Day 8) for stage and quality in accordance to the International Embryo Transfer Society (IETS) guidelines for bovine embryo assessment [29].

### 2.4 In vivo embryo collection

One month following the final session of Stimulated OPU, 8 of the original 9 heifers (one had become lame and was removed from the study) underwent estrous synchronization using the drugs and doses described in Section 2.2, and according to the following schedule: CIDR insertion on Day −10, PGF2_α_ on Day −4, CIDR withdrawal on Day −2, with estrus detected between Day −1 and Day 2. A second CIDR was inserted on Day 7 of the cycle, followed by GnRH (i.m.) on Day 9, and initiation of ovarian stimulation commenced on Day 11. This involved eight injections of FSH (Folltropin; i. m. every 12 h, reducing by 0.5 mL each day from 2.5 mL to 1 mL). PGF_2α_ (i.m.) was administered on the morning of Day 13, and artificial insemination (AI) commenced 48 h later, on Day 15, and was repeated three times at 12 h intervals.

Day 7 embryos were subsequently flushed transcervically following initial AI. Briefly, following light sedation (0.25 mL/100 kg i. m. xylazine hydrochloride; Rompun 2% w/v, Bayer Animal Health, Reading, UK) and epidural (adrenaline, lignocaine hydrochloride; 3–5 mL 2% w/v, Lignol, Dechra Veterinary Products, Shrewsbury, UK) anaesthesia, a Foley catheter was passed through the cervix and placed in each uterine horn. Three x 50 mL syringes containing Vigro™ Flush Media (Vetoquinol UK Ltd, Towcester, UK) was used to flush each horn. Flushes were passed over a 70 μM filter to recover embryos. Embryos were held in 2 mL of SOFaai [30] supplemented with 25 mM HEPEs media at 38 °C in transportable incubator until transported to the lab. A total of 21 embryos were retrieved from 5 donors. Recovered Day 7 *in vivo* derived blastocysts and morula were cultured for 24 h in SOFaai culture media [30] in 20 μL drops (up to 11 per drop, within donor) under oil at 5% CO_2_, 5% O_2_ and 38.5 °C, and subsequently graded as described for *in vitro* produced embryos.

### 2.5 Immunodissection, RNA and DNA extraction, and DNA amplification

A total of 336 (117 non stimulated, 219 stimulated) OPU-IVP derived and 20 (from same OPU donors) *in vivo* derived Day 8 blastocysts (IETS stages 6–9) underwent immunodissection to isolate the trophectoderm (TE) and inner-cell mass (ICM). Immunodissection was performed as described previously [31]. Briefly, the zona pellucida (ZP) was removed by 30–60 s exposure to 10 μg/mL pronase in TCM199. ZP free embryos were incubated for 60 min in 50% anti bovine sera in mSOF, washed, then incubated for 2 min 50% Guinea pig complement sera in mSOF. Each embryo was washed individually through PBS/PVP (Ca/Mg free PBS + 0.1% PVP), placed in a 5 μL drop of PBS/PVP under oil, and held on a warm stage for 40 min. Disaggregation was induced by pipetting using a fine-bore glass pipette. TE cells were transferred to a PCR tube containing 4 μL PBS and held on ice. The ICM was pipetted repeatedly to remove any adherent TE cells and the remaining ICM was then also transferred to a PCR tube. A sub-sample of immunodissected blastocysts (n = 75) were assessed for the expression of the TE specific marker *GATA3* [32] by qPCR (Supplemental Information Table S1). This confirmed good (P = 0.003) separation of the two blastocyst lineages (Supplemental Information Fig. S1). Remaining samples of TE and ICM were frozen at −80 °C until further analyses.

#### 2.5.1 Retrospective sample allocation

Nine ICM and nine TE of IETS Stage 7 (Grade 1 and 2), 8 and 9 (all Grade 1) blastocysts per donor, per treatment were selected for chromosomal analysis. Where 9 or less embryos were available, all Stages 7 (Grade 1 and 2), 8 and 9 were taken; if more than 9 embryos were available, blastocysts were randomly selected by stage in proportions that represented the stages exhibited by that donor for Stimulated and Non-stimulated cycles. A total of 82 blastocysts from Stimulated OPU cycles and 70 blastocysts from Non-stimulated were selected. All Stage 7 (Grade 1 and 2), 8 and 9 immunodissected *in vivo* derived (n = 20) blastocysts were selected.

### 2.6 DNA extraction

White blood cells (WBC) from whole blood of OPU donors were pelleted by centrifugation (2500 rpm for 3 min, followed by 14,000 rpm for 2 min). Semen samples (from the single sire used in these studies) were washed in PBS and pelleted by centrifugation at 14,000 rpm for 2 min. DNA was extracted from WBC and sperm using a salting-out method [33]. Briefly, cells were lysed by overnight incubation at 55 °C in 800 μL of 8 mM Tris/0.1 mM EDTA buffer with 0.01% (w/v) proteinase K and 0.5% (w/v) SDS; and for sperm cells only with 10 mM DTT. To reduce clumping 1.5 μL of 20 mg/mL proteinase K was added and lysed blood cells further incubated for 3 h at 55 °C. Following lysis, 360 μL of 5 M NaCl was added and solution centrifuged at 14,000 rpm for 5 min to remove cell debris. The DNA was then extracted from the supernatant by ethanol precipitation.

#### 2.6.1 Whole-genome amplification (WGA)

WGA used a REPLI-g single cell WGA kit (Qiagen, Manchester, UK) as per manufacturer’s instructions. Briefly, samples were denatured in a final volume of 40 μL denaturation buffer (supplied with kit) containing 75 mM DTT, by incubation at 65 °C for 10 min, which was ceased by the addition of 3 μL stop solution (supplied). WGA was achieved by the addition of 40 μL of master mix (supplied), containing Phi 29 polymerase, and incubation for 4 h at 30 °C, followed by 3 min at 65 °C to cease the reaction. Amplified DNA was stored at −20 °C until analyses.

#### 2.6.2 Quality assurance/sexing

All WGA DNA was submitted to PCR to assess amplification, to identify sex and to confirm concordance between TE and ICM lineages of individual blastocysts. Briefly, a multiplex PCR incorporated two primer sets: a primer set for *SRY* (GenBank accession no. EU58186.1 – designed in house using Primer-Blast (NCBI)) was used to identify male sex (TGAAACAAGACCAAAACCGGG forward, TCCATGGACTTGCTCTACTGT reverse; amplicon 339bp), and BSP [34] was used as an autosomal marker (TTTACCTTAGAACAAACCGAGGCAC forward, TACGGAAAGGAAAGATGACCTGACC reverse, amplicon 538bp). The PCR was carried out in a 25 μL reaction mix containing 12.5 μL of Immomix Red mastermix (Bioline Reagents Ltd, London, UK) and 0.05 μM each of the primer sets for BSP, 0.5 μM each of the primer set for *SRY*, and 1 μL of WGA DNA. The PCR program used an initial denaturation step at 95 °C for 10 min, then 30 cycles at 94 °C for 30 s, 55 °C for 30 s, and 72 °C for 60 s; a final cycle of 72 °C for 7 min then held at 10 °C.

### 2.7 Single nucleotide polymorphism (SNP) genotyping and chromosomal analysis

Whole genome amplified embryonic DNA (from both TE and ICM) and parental DNA were submitted to Neogen Europe Ltd (Ayr, Scotland, UK) for genotyping using a GGP 50K SNP array (Neogen Europe Ltd, UK). For this database, the average “Call Rate” (fraction of SNPs successfully genotyped) obtained was 95.8% ± 0.18%, and the average “Gene Call” (GC) scores (an estimate of the reliability of each genotype call) were GC50 = 0.747 ± 0.001, and GC10 = 0.424 ± 0.005. Raw data from the SNP output files for GC scores and signal strength measurements (X and Y) were employed to compile a full chromosomal analysis and to characterise the quality and origin (maternal/paternal and mitotic/meiotic) of aneuploidy. The tests employed included Karyomapping, B-allele frequency (BAF) and log R ratio (LRR) graphs, Gabriel-Griffin plots (GG-plots), and a mosaicism estimation model, all of which are further described below. During chromosomal analysis, operators were blinded with respect to the IVP protocol followed to produce the embryos.

#### 2.7.1 Karyomapping

Karyomapping was initially employed to detect many forms of aneuploidy (supplemental information, Table S2) and to identify the parental origin of these chromosome errors. Computations on the raw SNP database were performed on MobaXterm (version 11.1, build 3860, Mobatek). Information on individual embryo cell lineages plus their parents were extracted from the database. Individual samples were clustered by parental origin, and sibling embryos were analysed together in groups of three, resulting in six cell lineages (three TE and three ICM lineages) analysed together in the same output file. In each set, a cell lineage was selected to serve as a reference individual to permit Karyomapping haploblock tracing, as previously described [18]. The reference was later swapped to a different sample, to permit the analysis of all six cell lineages in each set. The analysis of the individual files was completed via the Microsoft Excel macro BoVision (version 3.1, University of Kent), as previously described [18]; however, the macro was upgraded to process multiple files at the same time. Whole chromosome errors were determined for all the samples in study. Chromosome Y and paternal chromosome X errors were excluded as these cannot be detected by Karyomapping (these chromosomes are present in single copy in the sire; haploblock tracing is thus prevented).

#### 2.7.2 Log R ratio (LRR) and B allele frequency (BAF) graphs

LRR and BAF graphs were used to further validate the diagnosis obtained by Karyomapping but also to provide a method to discriminate between loss of heterozygosity and monosomy and to detect certain trisomies of mitotic origin. The analysis pertaining to LRR and BAF graphs was conducted in R (R Core Team, 2014) and figures were produced using the package karyoploteR [35]. Samples identified as euploid by Karyomapping were used to calculate standard R and Theta values for each SNP combination (RAA, RAB, RBB, ThetaAA, ThetaAB, ThetaBB) from signal intensity data (X and Y) available from the SNP database itself. We employed the same R and Theta definitions recommended in Ref. [36]. These values (R and Theta) were then used to calculate the relevant data points for each LRR and BAF graph, as previously described [36]. A threshold was also employed so that only data points with GC > 0.60 were plotted.

#### 2.7.3 GG plots

GG plots were employed to clarify the origin (meiosis I, meiosis II, mitosis) of each trisomy detected by Karyomapping, or LRR/BAF methods. Computations on the raw SNP database as relevant to the production of GG-plots were performed in the same way described in Section 2.7.1Output files containing the appropriate information for each sample were processed through the VBA macro BoVisionGG (version 1.0, University of Kent). The GG-plots were drawn using the exact algorithm described in Ref. [37], and BoVisionGG was employed to automatically handle the drawing of multiple plots.

#### 2.7.4 Mosaicism

For each aneuploidy, mosaicism diagnoses at whole chromosome level were inferred by calculating the ratio between the average LRR values for the affected chromosome (LRRa) and the expected LRR value (LRRe) for the same chromosome when in monosomy or trisomy configuration (using the appropriate LRRe for either a monosomy or a trisomy). The LRRe values were calculated from the database in study by averaging all LRR values from aneuploid cases by chromosome and aneuploidy type (monosomy/trisomy); a variance threshold of <1 was implemented. A fixed and identical correction factor was added to both LRRa and LRRe values; this was equal to the difference between the average LRR of all euploid samples and 0. This factor was included to correct for the difference observed between the obtained LRR values and the theoretical average LRR value for euploid samples (zero). The ratio LRRa/LRRe was then used as a simple way to provide an indication for the presence and percentage of mosaicism for each chromosome abnormality previously detected (by Karyomapping, LRR and/or BAF analysis). In accordance with the suggestions made by Ref. [38], chromosomal errors presenting ratios >0.8 were classified as being in a non-mosaic configuration (affecting the whole cell lineage), whilst errors with ratios between 0.2 and 0.8 were considered mosaic; the ratio itself providing an indication for the proportion of cells affected by the mosaic phenotype. All calculations were performed in R.

### 2.8 Statistical analysis

Analyses were performed using the GenStat statistical package (19th Edition, VSN International, 2018; https://www.vsni.co.uk/). All proportion data were analysed using REML generalized linear mixed models that assumed binomial errors and used logit-link functions. In these models ‘Donor’ formed the random model when testing the effects of ‘Treatment’ (i.e. stimulated vs non-stimulated) which was added as a fixed effect. Blastocyst ‘Stage/Grade’ was also added as a fixed effect in some models of chromosomal abnormalities. These anlayses of chromosomal abnormalities were initially undertaken for trophectoderm (TE) and inner-cell mass (ICM) lineages separately (to avoid inflating degrees of freedom). ‘Lineage’ (i.e. trophectoderm vs inner-cell mass) was subsequently added as a fixed effect to these models. Analyses of the number of follicles aspirated, oocytes retrieved and chromosome error classification assumed Poisson errors and used log-link functions. Data are presented as means ± SEM. Follicle size distribution was analysed using chi-square tests.

## 3 Results

In total, 783 COCs (424 from stimulated and 359 from non-stimulated cycles) were retrieved by OPU of which proportionately around 0.88 were inseminated following IVM. This generated 459 Day 8 blastocysts of which 152 of the more advanced stages (IETS 7–9) were chosen for analysis of chromosome copy number. In addition, we were successful in recovering and analysing 20 *in vivo* produced embryos from five of the same donors.

### 3.1 Ovarian stimulation significantly improves all measures of IVP

All measures of oocyte retrieval and IVP were improved through the use of ovarian stimulation (coasting protocol) (Table 1). Follicle diameter at the point of aspiration was greater (P < 0.001) for stimulated than non-stimulated cycles (supplemental information, Fig. S2). The number of follicles aspirated, oocytes retrieved per cycle and per follicle were all also greater (P < 0.001) for the stimulated cycles (Table 1). Similarly, the proportion of high-quality COCs (Grades 1 and 2) was improved (P < 0.001). By Day 2 following insemination, the proportion cleaved of inseminated was marginally (i.e. 0.945 vs 0.887) greater (P < 0.001) for stimulated than non-stimulated cycles; the same marginal improvement was also evident with regard to the developmental stage of zygotes. Ultimately, Day 8 blastocyst yields and quality (based on morphological grade) were enhanced (P < 0.001) for oocytes derived from stimulated relative to non-stimulated cycles.

**Table 1:** Effect of ovarian stimulation on oocyte recovery and *in vitro* embryo production over three stimulated cycles preceded by six non-stimulated of transvaginal follicular aspiration (OPU). All expected measures of improvement favour the stimulated (with coasting) protocol.

	Stimulated (coasting)	Non-stimulated	P
Number of cycles	3	6	
Aspiration			
Number of follicles aspirated per cycle	27.3 ± 1.31	15.0 ± 0.62	<0.001
Number of oocytes retrieved per cycle	19.5 ± 1.1	7.9 ± 0.44	<0.001
Number of oocytes retrieved per follicle	0.710 ± 0.0182	0.526 ± 0.0174	<0.001
Oocyte Culture			
Number of Oocytes matured per cycle	18.6 ± 0.91	6.7 ± 0.35	<0.001
Proportion of Grade 1 COCs	0.391 ± 0.0232	0.118 ± 0.0155	<0.001
Proportion of Grade 2 COCs	0.258 ± 0.0208	0.186 ± 0.0187	0.01
Proportion of Grade 3 COCs	0.310 ± 0.0220	0.499 ± 0.0241	<0.001
Proportion of Grade 4 COCs	0.041 ± 0.0094	0.197 ± 0.0192	<0.001
Day 2 cleavage-stage embryos			
Number of oocytes inseminated per cycle	17.9 ± 0.88	6.5 ± 0.36	<0.001
Proportion of inseminated oocytes cleaved	0.945 ± 0.0124	0.887 ± 0.0197	0.017
Proportion at 2 cell stage	0.017 ± 0.0064	0.078 ± 0.0155	0.001
Proportion at 3–4 cell stage	0.110 ± 0.0200	0.140 ± 0.0260	NS
Proportion at >4 cell stage	0.872 ± 0.0223	0.782 ± 0.0332	0.019
Day 8 blastocysts			
Proportion of inseminated oocytes	0.712 ± 0.0288	0.466 ± 0.0360	<0.001
Proportion of cleaved zygotes	0.753 ± 0.0272	0.525 ± 0.0369	<0.001
Proportion reaching Stage^a^ 8 or 9	0.561 ± 0.0325	0.370 ± 0.0440	<0.001
Number reaching Stage^a^ 8 + 9 per cycle	7.2 ± 0.65	1.1 ± 0.17	<0.001
Number reaching Stage^a^ 7(1&2)^b^ to 9 per cycle	11.1 ± 0.92	2.2 ± 0.27	<0.001

a IETS scale [29].

b (morphological grade).

### 3.2 Chromosomal abnormalities are not affected by ovarian stimulation but decline during embryo development and differ between donors

**Fig. 1:**
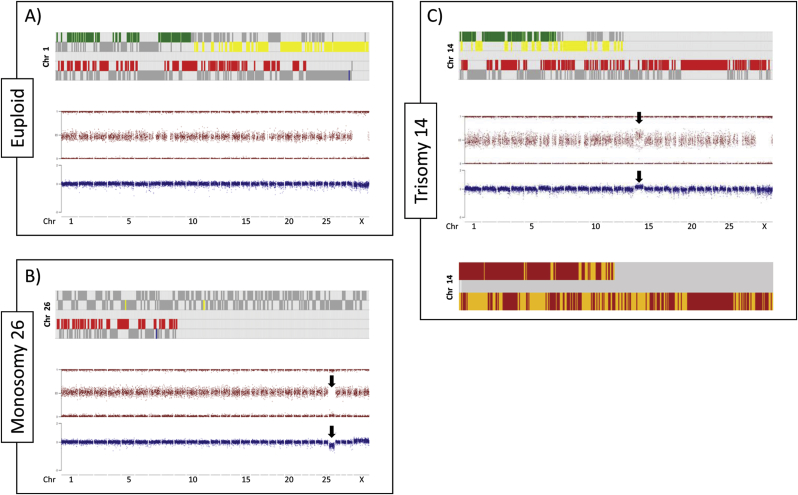
Chromosome analysis outputs of bovine blastocysts. Each aneuploidy was analysed by multiple algorithms: Karyomapping, Signal strength intensity metrics (BAF and LRR) and, in the case of trisomies, Gabriel-Griffin plots. With Karyomapping, a diagram for each chromosome illustrates which haploblocks the embryo inherited from its dam (yellow/green) and sire (blue/red). Euploid chromosomes are identified by few, continuous blocks of yellow/green or blue/red bands, a pattern that becomes altered in aneuploidy resulting in excessive banding in trisomies and absent haploblock inheritance in monosomies. Signal strength intensity graphs plot the signal strength intensity of SNP alleles A and B relative to each other (for BAF plots) or combined (in LRR plots). Euploid chromosomes display 3 signal clusters in a BAF plot according to each genotype (BB = 1, AB = 0.5, AA = 0) and a single consistent cluster of data close to the average expected signal intensity in LRR the plots. Monosomies appear as a loss of the AB cluster in the BAF graph and a decrease in signal intensity in the LRR graph for a specific chromosome; conversely, trisomies result in the gain of an additional signal cluster in a BAF graph (for the possible allele combinations BBB, ABB, AAB, AAA) and an increased signal intensity on the LRR graph for the specific chromosome. In Gabriel-Griffin Plots, for each trisomy, parental haploblocks are defined and a diagram is drawn highlighting heterozygous genotype calls in red and homozygous calls in yellow. Trisomies originating in Meiosis I show an uninterrupted heterozygous pattern around the centromere, Meiosis II error are identified by yellow blocks around the centromere, and mitotic errors are characterized by a variegated yellow and red pattern across the chromosome. **(A)** Top = Euploid Karyomapped chromosome 1; Middle (in red) and Bottom (in blue) = BAF and LRR graphs (respectively) also demonstrating an euploid karyotype. (**B**) Top = Monosomy 26 of maternal origin, note the absence of any yellow/green (maternally inherited) haploblocks; Middle (in red) and Bottom (in blue), BAF and LRR graphs (respectively) demonstrating the same monosomy (arrows). **(C)** Top = Trisomy 14 of maternal origin, note the excess yellow/green banding; Middle (in red) and Bottom (in blue) graphs = BAF and LRR graphs (respectively) demonstrating the same trisomy (arrows); Bottom most = Gabriel-Griffin plot suggesting this trisomy originated in the maternal germline during Meiosis I (due to the prominent red pattern encompassing the top two thirds of chromosome 14 and, therefore, its centromere).

**Fig. 2:**
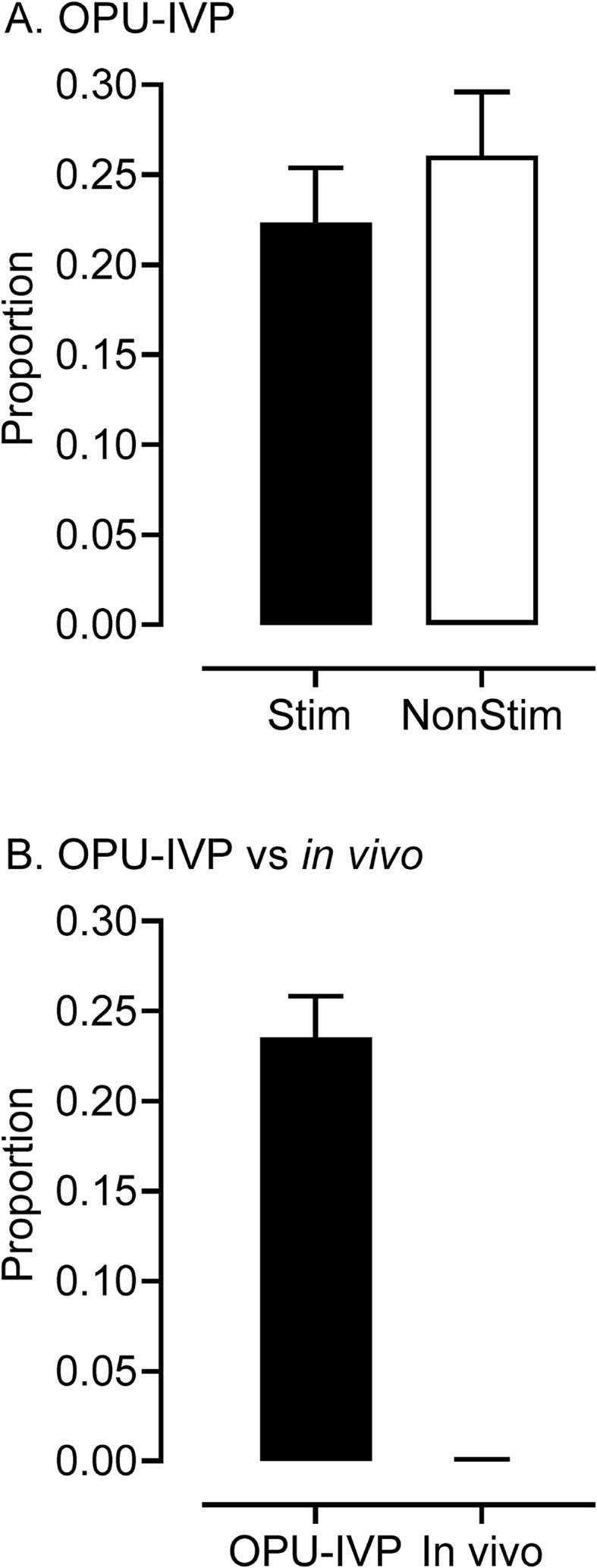
Incidence of aneuploidy did not differ between Day 8 blastocysts generated from stimulated (n = 82 blastocysts) vs non-stimulated (n = 70 blastocysts) cycles of OPU (A) but, in matched donors, did differ (P = 0.004) in Day 8 blastocysts derived from cycles of OPU-IVP compared to ovarian-stimulation, artificial insemination and embryo recovery (*in vivo*) (B).

The proportion of embryos selected for aneuploidy screening that were male averaged 0.592 ± 0.0398 and did not differ between ovarian stimulation treatments. Example plots (based on Turner et al. [18]) for euploid and aneuploid cases are presented in Fig. 1. The overall incidence of aneuploidy for these 152 OPU-IVP Day 8 (IETS stage 7–9) blastocysts was 0.241 ± 0.0231 and this value did not differ significantly between stimulated and non-stimulated cycles (Fig. 2A). This indicates that ovarian stimulation followed by coasting does not increase the levels of aneuploidy *per se.* Comparison to *in vivo* derived blastocysts (matched for the five common donors from which these embryos were collected) did, however, reveal a significant (P < 0.001) difference. That is, all 20 *in vivo* derived blastocysts were chromosomally normal (euploid) (Fig. 2B). Given that these *in vivo* derived blastocysts also originated from stimulated cycles, this observation further points to IVM/IVC as the primary cause of chromosomal abnormalities.

**Fig. 3:**
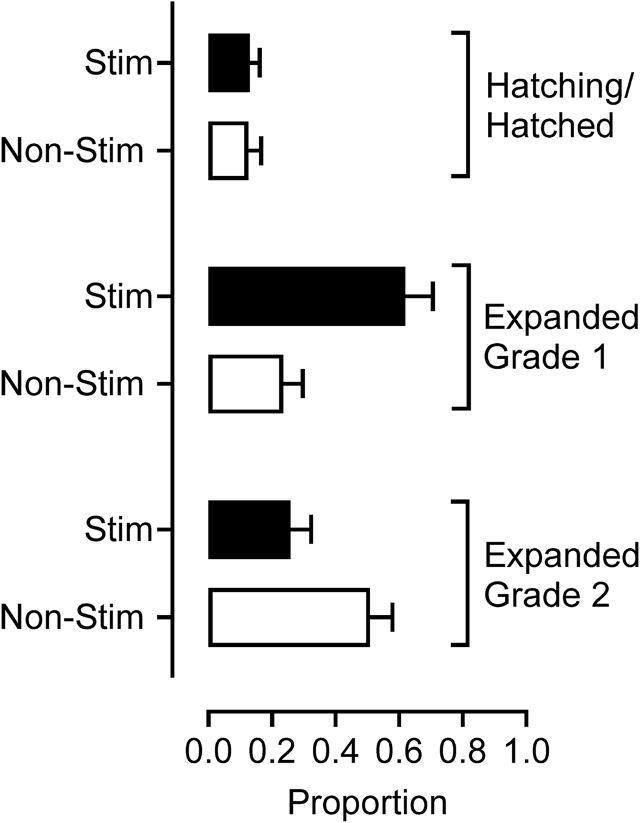
Incidence of aneuploidy in Day 8 blastocysts from stimulated (n = 82 blastocysts) and non-stimulated (n = 70 blastocysts) cycles of OPU. Data represent proportion aneuploid Grade 1 hatched/hatching blastocysts, and Grade 1 and 2 expanded blastocysts. The proportion aneuploid was less (P < 0.01) for Stage 8/9 (hatching/hatched) blastocysts compared to Stage 7 (expanded) blastocysts. However, there was an interaction (P < 0.05) between ovarian-stimulation treatment and blastocyst stage/grade. Aneuploidy decreased as stage/grade improved for blastocysts from non-stimulated cycles but was greatest for expanded Grade 1 blastocysts from stimulated cycles.

The incidence of chromosomal abnormalities was generally lower (P < 0.01) in the developmentally more advanced Day 8 blastocysts (IETS Stage 8/9) compared to less advanced Stage 7; and in better quality embryos (morphological Grade 1 vs Grade 2) as shown in Fig. 3. However, there was an interaction (P < 0.05) between ovarian-stimulation treatment and blastocyst stage/grade. This indicated that, although the incidence of chromosomal abnormalities decreased as stage/grade improved for blastocysts from non-stimulated cycles, curiously it was greatest for expanded (Stage 7) Grade 1 blastocysts from stimulated cycles.

**Fig. 4:**
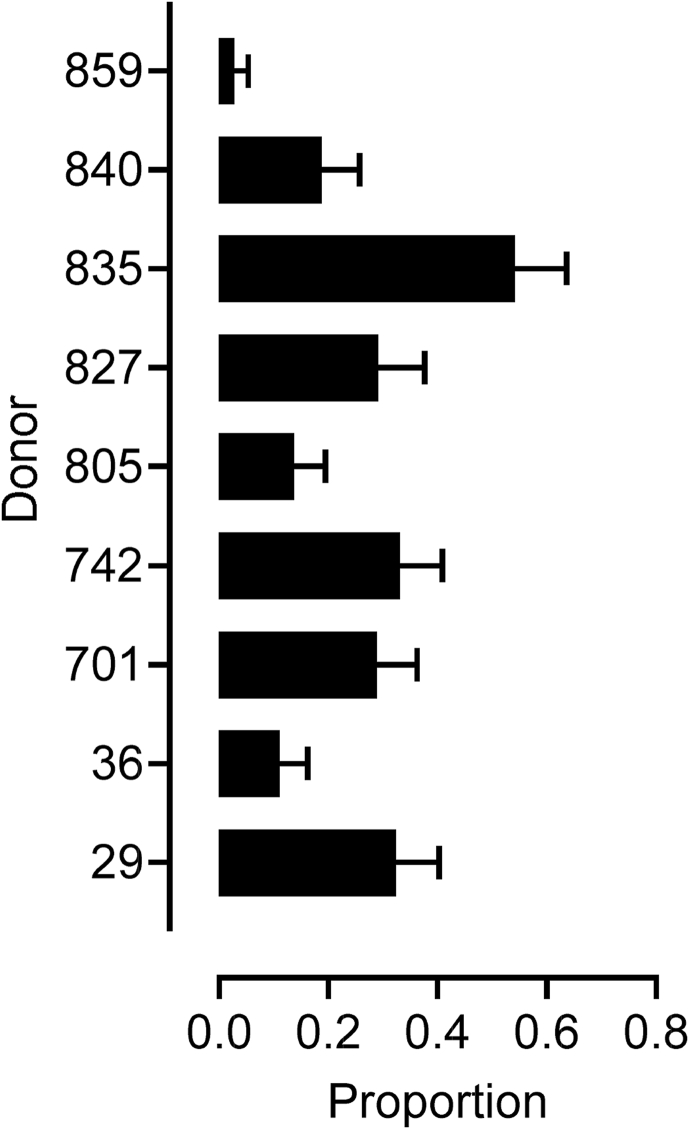
Incidence of aneuploidy in Day 8 blastocysts (all IETS Stage 7–9 blastocysts; n = 152) per donor averaged across stimulated and non-stimulated cycles. The proportion of aneuploid blastocysts differed (P = 0.008) between donors.

In contrast to the overall effects of ovarian stimulation, there was a marked (P = 0.008) difference in the proportion of chromosomally abnormal blastocysts between donors (Fig. 4). That is, virtually all blastocysts from Donors 805 and 859 were euploid, whereas a relatively high proportion of blastocysts from Donor 835 were aneuploid.

### 3.3 Chromosomal abnormalities were predominantly maternal meiotic aneuploidy or (hypo)triploidy

**Fig. 5:**
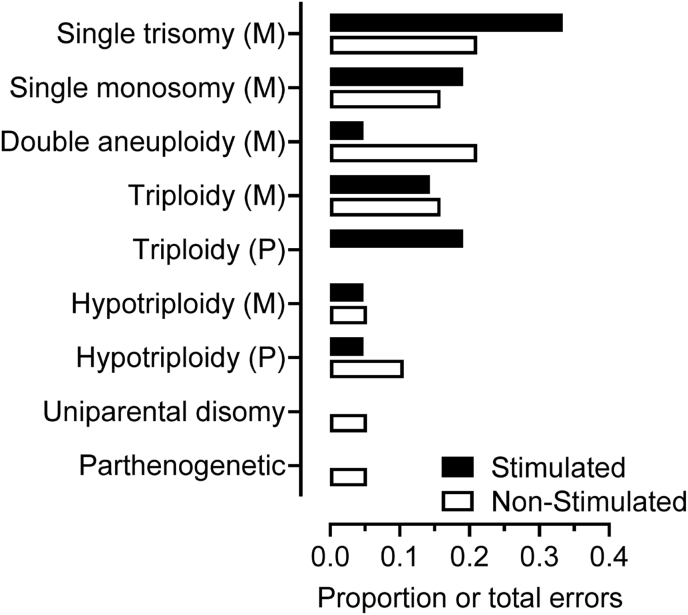
Nature of chromosomal errors expressed as a proportion of total errors in Day 8 blastocysts (n = 40). These did not differ significantly between stimulated (■) and non-stimulated (□) cycles of OPU. ‘(M)’ and ‘(P)’ refers to maternal and paternal origin respectively. ‘Double aneuploidy’ refers to combinations of maternal trisomy and/or maternal monosomy on two chromosomes.

A total of 40 OPU-IVP blastocysts were found to have an abnormality in at least one lineage. Abnormalities largely sub-divided into aneuploidy (including double aneuploidy) and triploidy (including hypotriploidy) (Fig. 5). A total of 11 (proportionately 0.28) were single maternally derived trisomies and 7 (∼0.18) were single maternal monosomies. Five (0.125) were double aneuploidies (a double trisomy, a double monosomy and three trisomy/monosomy). We observed no single or double paternally-derived aneuploidies. A total of 5 (0.125) were triploid (4 maternal, 1 paternal) and 2 hypotriploid (2 maternal, 0 paternal), with a single embryo having only a maternal genome indicating parthenogenesis. All of the above had the same karyotype in ICM and TE.

Of the remaining 9 (∼0.23) non-concordant embryos, four were triploid in one lineage and euploid in the other, and one was triploid in the TE and parthenogenetic in the ICM. One blastocyst was euploid in the TE and exhibited UPD in the ICM. The three remaining blastocysts were paternal hypotriploids, two of which were euploid in one lineage; the other had predominantly paternal chromosomes (two of which were lost in the TE) with evidence of persistent maternal chromosomes in the ICM. However, the overall incidence of specific categories of chromosomal error did not differ between blastocysts generated from stimulated and non-stimulated cycles.

**Fig. 6:**
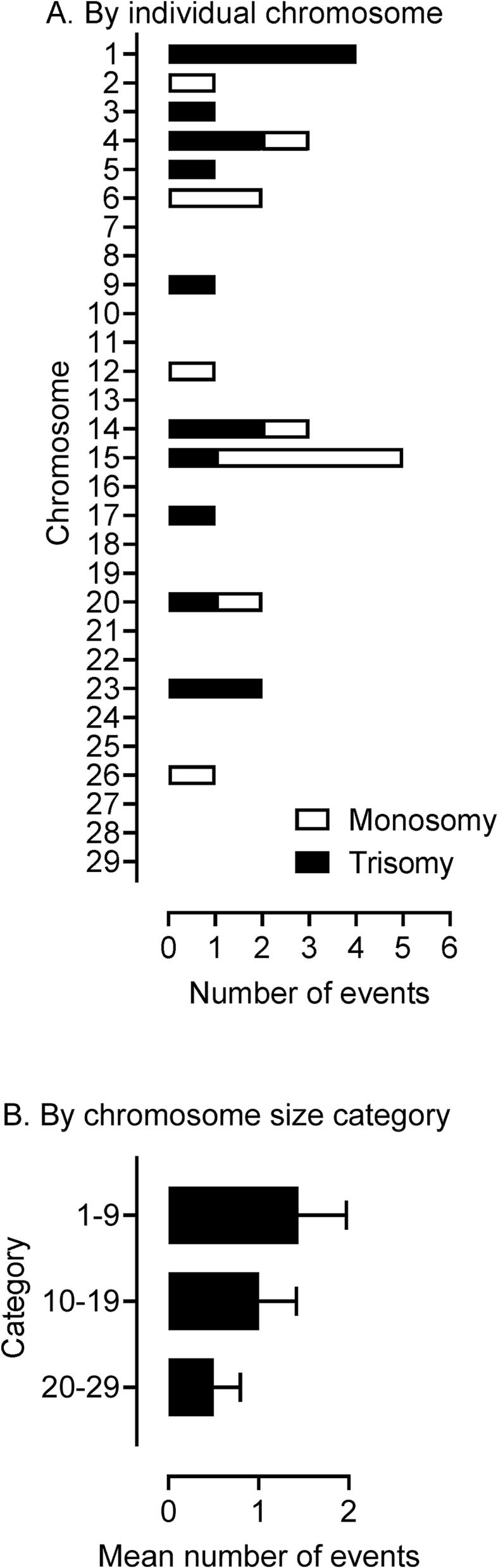
Chromosome-specific incidence of aneuploidy in blastocysts (n = 152). (A) Expressed by individual chromosome identifying incidences of monosomy and trisomy. (B) Combined aneuploidy categories grouped by ‘small (20–29), medium (10–19) and large (1–9)’ chromosomes. Data are for blastocysts from stimulated and non-stimulated cycles of OPU-IVP.

Although it was not possible to analyse formally errors on a chromosome-by-chromosome basis, due to small numbers, chromosome 15 (5x) plus chromosome 1 (4x), and 4 and 14 (3x each), were particularly over-represented (Fig. 6A). The incidence of aneuploidy appeared greater for the larger autosomes (i.e. chromosomes 1–9), although this did not reach statistical significance (Fig. 6B).

### 3.4 Broad concordance between ICM and TE lineages

**Fig. 7:**
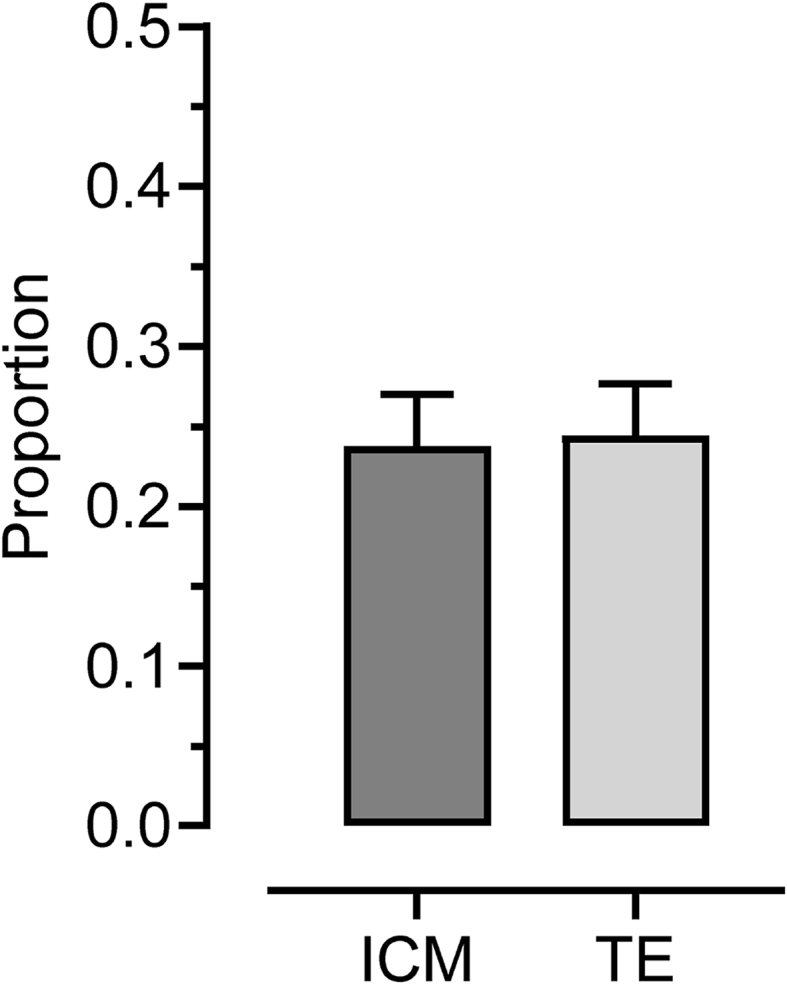
Incidence of aneuploidy for the inner-cell mass (ICM) and trophectoderm (TE) cell lineages for blastocysts derived from stimulated (n = 82 blastocysts) and non-stimulated (n = 70 blastocysts) cycles of OPU-IVP.

**Fig. 8:**
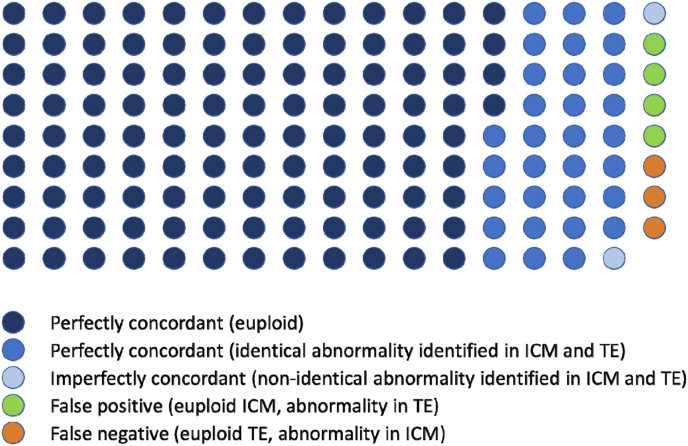
Depiction of the 152 OPU-IVP blastocysts analysed for chromosome abnormalities in both ICM and TE lineages. The perfect concordance of 112 euploid and 31 abnormal embryos indicates an incidence proportionately of 0.94. This increases to 145 (proportionately 0.95) for perfect and imperfect concordance combined. The 4 so called “false positives” should, most likely, not be transferred as they may lead to placental insufficiency, leaving only 3 (proportionately 0.02) in which a genuine misdiagnosis (false negative) was made.

The incidence of aneuploidy was similar for both the ICM and TE lineages (Fig. 7). Moreover, of the 152 OPU-IVP embryos (82 stimulated, 70 non-stimulated) assessed for chromosome abnormalities in both cell lineages, 143 (proportionately 0.94) were perfectly concordant; that is the diagnosis was identical in the TE and ICM (Fig. 8). Of the remaining 9 (0.06), 2 (0.01) were “imperfectly concordant”; that is a dissimilar abnormality was observed in the TE and ICM (so a TE biopsy would detect an error). This left 7 (0.05) with a discordant result (normal in one lineage but abnormal in the other), of which 4 (0.03) would have been a “false positive” in a diagnostic setting (i.e. a TE biopsy would have incorrectly indicated an abnormal embryo) and 3 (0.02) a “false negative” (i.e. a normal result returned for an abnormal embryo). Of the 9 “not perfectly concordant” returns, 5 were from stimulated and 4 from non-stimulated cycles (not statistically significant).

Signal intensity analysis allowed mosaic embryos with between 20 and 80% mosaicism to be identified. Five of the mosaic embryos were predominantly aneuploid (4 trisomy, 1 monosomy) with some euploid contribution in one or both lineages. Of the trisomies, one was mosaic in both the TE and ICM, two mosaic only in the ICM and, one mosaic only in the TE. The monosomy was mosaic only in the TE. For all these samples, the aneuploidy was estimated to affect >50% of the sample (range from 53 to 77%). A single predominantly euploid embryo was identified as mosaic for UPD restricted to the ICM, but it was not possible to ascertain the proportion of the sample affected.

## 4 Discussion

A number of key findings emerge from the current study. We accept our first hypothesis that ovarian stimulation leads to a significant increase in the yield of high-quality oocytes and potentially transferrable blastocysts, but not at the expense of an increase in aneuploidy. Our second hypothesis that there is strong concordance between the aneuploidy diagnosis made from the TE and that of the ICM lineage is also accepted. Our incidental findings are that levels of aneuploidy are lower in developmentally more advanced and morphologically better-quality blastocysts, that there is a significant degree of variability in the incidence of aneuploidy between oocyte donors, and that there is evidence that some chromosomes are more prone to non-disjunction in the oocyte.

### 4.1 Stimulated vs non-stimulated cycles

Ovarian stimulation is commonly practiced in *Bos taurus* cattle undergoing OPU although there is increasing interest in reducing the level of hormonal interventions in these genotypes. In the current study, the ‘coasting’ ovarian stimulation protocol led to a 5- to 7-fold increase in the yield of developmentally advanced and morphologically good quality Day 8 blastocysts per donor cycle over the non-stimulation protocol (Table 1). This improvement in yield was greater than that which could be achieved with the increased frequency (4 vs 1) of non-stimulated cycles over a standard 14-day period. The benefits of ovarian stimulation over non-stimulation would be enhanced further if these embryos led to improved pregnancy outcomes following ET. This is the subject of ongoing investigations at our collaborating laboratories.

With the foregoing discussion in mind, it is important to emphasize that the observed improvement in blastocyst yield was not at the expense of an overall increase in aneuploidy. That is, the incidence of chromosomal abnormalities (at ∼0.24 of embryos tested) was not elevated when ovarian stimulation (with coasting) was employed. It is notable, however, that there were some differences in the nature of chromosomal errors between stimulated and non-stimulated cycles. The incidence of ‘double errors’, which consisted combinations of maternal trisomy and/or monosomy on two separate chromosomes, was numerically greater in blastocysts from non-stimulated cycles. Conversely, the incidence of maternal trisomy on single chromosomes was greater in blastocysts from stimulated cycles (Fig. 5). In contrast to OPU-IVP blastocysts, all our *in vivo* derived embryos from the same donors were euploid. Although caution is advocated when interpreting this observation (given their relatively low numbers; n = 20 *in vivo* derived embryos), the highly significant difference, and the predominance of maternal meiotic errors, implicates the 24-h culture period between OPU and insemination as the time point at which most abnormalities arise.

### 4.2 Effect of stage and grade

The overall incidence of chromosomal abnormalities was lower in the developmentally more advanced Day 8 blastocysts (proportionately 0.128 vs 0.373 for hatched/hatching vs expanded) indicating an association between advanced development and aneuploidy levels. Although blastocyst stage and grade are generally considered poor indicators of euploidy in human embryos [39,40], several studies have reported associations between morphological grade and the incidence of chromosomal abnormalities in both cleavage- [[41], [42], [43], [44], [45]] and blastocyst-stage [39,[46], [47], [48], [49], [50]] embryos. In contrast, the very few reports describing such associations in bovine embryos are conflicting and reliant on superseded technologies [51,52]. In the current study, the declining incidence of chromosomal errors with morphological stage is in line with studies reporting arrest [53] or reduced rates of progression [54] linked to chromosomal errors in human embryos. For morphological grade, we observed some qualitative differences in that monosomies occurred more often (∼70%) in lower grade expanded blastocysts, whereas trisomies were the predominant error observed in higher grade expanding, hatching and hatched blastocysts; a pattern also observed in human embryos [47].

The increase in aneuploidy in Grade 1 expanded (Stage 7) blastocysts derived from stimulated cycles (Fig. 3) is of interest. These errors consisted mostly of maternal trisomies on single chromosomes which, in human embryos, don’t associate with adverse morphology [50] and are generally considered to be more compatible with development [55]. In contrast, chromosomally abnormal Stage 7, Grade 2 blastocysts were mostly monosomic or were triploid/hypotriploid; and these errors are more likely to associate with adverse morphologies [47,48] and to be embryonic lethal [56]. Reasons for this association are unclear, but if it could be corroborated, then it would have important implications for blastocyst selection from stimulated cycles based solely on morphological criteria. We have yet to establish, however, if any of these differences might affect pregnancy outcomes following ET and this will form the basis of future studies.

### 4.3 Individual specific rates of aneuploidy among donors

It was evident that there was a high degree of variability in the incidence of chromosomal errors between individual donors. To the best of our knowledge there have been no convincing reports of genetic associations of elevated aneuploidy. These may be resolved by genome-wide association studies, should a sufficiently large number of samples such as these become available.

A number of intriguing observations also arise in this context when comparing stimulated vs non-stimulated cycles. For donors 859, 805 and 36, chromosomal errors were detected in blastocysts derived almost exclusively from non-stimulated cycles. Likewise, the incidence of chromosomal errors was less in stimulated than non-stimulated cycles for 827 and 701. In contrast, it increased with ovarian stimulation for donors 29, 835 and 840. Although variable donor responses to ovarian stimulation, in terms of blastocyst yields, are well documented in both cattle [57] and humans [58], to the best of our knowledge, the relationship between ovarian stimulation regimen and chromosomal error rate between individual donors is not known. Furthermore, whilst early animal [[59], [60], [61]] and subsequent human [62,63] studies reported deleterious effects of ovarian stimulation, more recent stimulation protocols do not appear to increase the incidence of chromosomal errors [[64], [65], [66]]. Donor variation in the nature/incidence of aneuploidy following ovarian stimulation clearly merits further investigation.

### 4.4 Nature and incidence of aneuploidy

The prevalence of any one given chromosomal error is a balance between the competing factors of initial error rate in the gamete and differential survival at any one given stage. For instance, an extra chromosome 16 is the most common of all trisomies in humans but is not seen at all in live births as it invariably leads to spontaneous abortions. Despite obvious evidence of selection in humans, the consensus is that smaller chromosomes such as 16, 21 and 22 at least are more prone to non-disjunction and that most monosomies are lost before a pregnancy is clinically recognised [67]. In contrast to humans, the larger of the cattle chromosomes in the current study appeared to be more prone to nondisjunction (Fig. 6). The number of monosomies and trisomies also appeared to be particularly high for chromosomes 14 and 15. The reasons for these apparent species differences are unclear and require confirmation by further studies. Nevertheless, the numerically greater number of trisomies than monosomies (Fig. 5), and the fact that our assay detects only meiotic trisomies but monosomies of both meiotic and mitotic origin, suggest that the more lethal monosomies have been selected against by the blastocyst stage.

### 4.5 Concordance between TE and ICM lineages

The high degree of perfect concordance in the incidence (proportionately 0.94) of chromosomal errors between the TE and ICM (Fig. 8) is punctuated by nine non-concordant cases. Of these, two demonstrated that there was clearly an error in both lineages (imperfect concordance). The first embryo had a triploid TE but only maternal chromosomes in the otherwise diploid ICM, a presumed mechanism of complete loss of the paternal genome, confined to the ICM and therefore occurring in the very early cleavage divisions. The second had only paternal chromosomes (less chromosomes 19 and 29 which were lost in the TE) but with traces of the maternal genome in the ICM. This suggests loss of the maternal genome in most of both lineages, again relatively early in cleavage and perhaps independent post-zygotic loss of chromosomes 19 and 29.

Of the false positive results, all had a triploid or hypotriploid TE that would suggest that, although the ICM was chromosomally normal, the chances of a normal ongoing pregnancy and live birth would be minimal because of insufficiency of the chromosomally abnormal placenta. Again, a mechanism of loss of the extra genome is presumed. If we accept that the broader purpose of PGT-A is primarily to spot an abnormality (and hence inform whether or not to proceed with embryo transfer) we can be confident that PGT-A was informative for these cases also. Two of the remaining false negatives failed to identify an otherwise triploid embryo that would fail to develop; the final false negative was a UPD; an effective “misdiagnosis rate” of only 2%.

The presence of an abnormality in either the ICM or the TE is an example of confined mosaicism and has been extensively reported for human embryos [25,68]. Moreover, the high degree of concordance we observed is near identical to that observed in human studies [25,26]. This might be expected for chromosomal errors of meiotic origin that are clonally propagated [53]. Destouni et al. [69] reported that paternal chromosomal errors observed in bovine cleavage stage embryos occur as mosaics and are the result of dispermic fertilization, which can also lead to triploid and complex aneuploid mosaics in human embryos [70]. Our results indicate that (hypo)triploid embryos can have an extra maternal (diploid egg) or paternal (dispermy or, less likely, diploid sperm) origin in roughly equal numbers. The fact that we observed triploid/diploid mosaics and, when chromosomes were lost in the hypotriploid embryos, they were exclusively from the parent that donated the extra genome, suggests active mechanisms to evict the offending chromosomes very early in development (e.g. around fertilization or first cleavage). Alternative animal models (non-human primates and mice) have demonstrated that cells from one lineage with chromosomal errors can be preferentially removed by fragmentation [71] or by apoptosis which leads to disproportionate presence in the other [72].

It is noteworthy that we did not detect any blastocysts that we might describe as “chaotic” (i.e. multiple errors, different in each lineage of both number and structure). These are well described in human embryos but, had we seen them, we would have expected extensive loss of chromosomes from both parents together (not just one or the other), a higher degree of structural abnormality and a greater level of mosaicism. Our combined SNP-array analyses (based on Karyomapping, G-G and BAF/LRR plots) of ICM and TE cells was able to detect aneuploidies of both meiotic and mitotic origin, albeit not at cell-to-cell level [15]. Errors of mitotic origin constitute the predominant source of chromosomal mosaicism in the human preimplantation embryo [73]. Using a SNP-array based procedure not dissimilar to that employed in the current study, but on single blastomeres from cleavage-stage (Day 2–3) bovine embryos, Tsuiko et al. [74] observed a higher incidence of chromosomal abnormalities following stimulated cycles of oocyte recovery and *in vitro* culture (OPU-IVP) compared to stimulated cycles with *in vivo* embryo recovery. This is in keeping with observations from the current study indicating that IVP, rather than ovarian stimulation, is the major cause of chromosomal errors in ART. Embryos in the study of Tsuiko et al. [74], however, displayed a broad range of whole chromosome, segmental and ploidy aberrations which is consistent with the high incidence (15–90%) of mosaicism observed in cleavage stage human embryos [73]. Mosaicism generally decreases by the blastocyst stage in humans, leading Fragouli et al. [53] to conclude that TE biopsy for PGT-A is likely to provide a more reliable indication of cytogenetic status. Overall, therefore, our observations support the use of PGT-A in TE biopsies from embryos undergoing genomic evaluation in commercial cattle breeding.

### 4.6 Concluding remarks

The current study demonstrates that stimulated ovarian cycles (with ‘coasting’) prior to oocyte collection (OPU) leads to greater yields of blastocysts without compromising chromosome integrity. It suggests that PGT-A may be actively employed in cattle breeding, particularly as we have established that trophectoderm biopsies represent the ploidy status of the overall embryo. This is significant as it means that our contemporary SNP-array based platform can be used to undertake genomic evaluations (gEBVs) and PGT-A assessments (for future pregnancy outcome evaluations) in bovine blastocysts simultaneously. One of the criticisms of human PGT-A is that the embryo biopsy procedure may compromise the developmental potential of the embryo thereby negating any positive effect gained from aneuploidy screening. In cattle embryos, given that the primary purpose of biopsy is to assess gEBV by SNP array, PGT-A can only confer an advantage. Consistent with the results of Tsuiko et al. [74], our finding that *in vitro* maturation/culture rather than ovarian stimulation *per se* is the primary cause of chromosomal abnormalities in pre-implantation embryos offers hope for future improvement. In theory, improvement in culture conditions to mimic more closely the *in vivo* environment could help reduce chromosome abnormalities further, particularly if donors that are prone to higher levels of error (as revealed in this study) are eliminated from the breeding programme. Finally, our data provide fundamental insight into the mechanisms of the origin of chromosome error in early development.

## Funding

This work was supported by the 10.13039/501100000268Biotechnology and Biological Sciences Research Council (10.13039/501100000268BBSRC) LINK awards scheme (BB/R007985/1; BB/R00708X/1). GG-A. was in receipt of a scholarship from the Ministry of Education, Turkey.

## Declaration of interest

The authors have no conflicts of interest to declare.

## Data statement

Data can be made available upon reasonable request.

## CRediT authorship contribution statement

**D.A.R. Tutt:** Investigation, Formal analysis, Writing - original draft, Writing - review & editing. **G. Silvestri:** Methodology, Formal analysis, Writing - review & editing. **M. Serrano-Albal:** Formal analysis. **R.J. Simmons:** Investigation, Writing - review & editing. **W.Y. Kwong:** Methodology, Investigation. **G. Guven-Ates:** Investigation, Writing - review & editing. **C. Canedo-Ribeiro:** Formal analysis. **R. Labrecque:** Resources, Writing - review & editing. **P. Blondin:** Resources, Writing - review & editing. **A.H. Handyside:** Software, Methodology. **D.K. Griffin:** Funding acquisition, Conceptualization, Methodology, Formal analysis, Writing - review & editing. **K.D. Sinclair:** Funding acquisition, Conceptualization, Resources, Project administration, Investigation, Formal analysis, Writing - original draft, Writing - review & editing.
