# Supplementary material for: Analysis of bovine blastocysts indicates ovarian stimulation does not induce chromosome errors, nor discordance between inner-cell mass and trophectoderm lineages
Source: Theriogenology. 2021 Feb;161:108–19. doi: 10.1016/j.theriogenology.2020.11.021 (PMC7837012; doi:10.1016/j.theriogenology.2020.11.021)
Supplement: Multimedia component 1 [file mmc1.docx]

**Supplementary Data**

**Validation of cell-lineage purity**

This was based on transcript expression for the trophectoderm (TE) cell marker GATA Binding Protein 3 (*GATA3*). Primers for *GATA3* and eight reference genes were designed using Primer Express software version 3.0.1 (Applied Biosystems) and were supplied by Eurofins Genomics (Ebersberg, Germany) (Table S1). Primers were tested using bovine liver cDNA and PCR products were sequenced by Source Bioscience (Nottingham, UK). Data were normalised to the four most stable reference genes (*YWHAZ*, *TBP*, *H2AFZ*, *B2M*) using Reference Gene Selector tool in CFX Maestro™ Software based on geNorm algorithm. Reference gene selection was confirmed using geNorm software based on geNorm M and V values.

Poly A+ RNA was extracted from pooled (n=15) inner-cell mass and trophectoderm lineages using Dynabeads® mRNA DIRECT™ purification kit (Invitrogen Ltd., Paisley, UK), following the manufacturers protocol, and complementary DNA (cDNA) synthesised using the QuantiTect® Reverse Transcription kit (Qiagen Ltd., address). The qPCR reaction contained 10 μl QuantiNova® SYBR® Green (Qiagen Ltd.), 1 μl of each primer, 2 μl of cDNA template and 6 μl RNase-free water. A negative no template control sample was run for each tested primer set using water instead of cDNA template, and minus reverse transcription (–RT) controls were tested to confirm the absence of gDNA. Each 20 μl sample was analysed in duplicate using low profile, non-skirted, clear 96-well plates in the Bio-Rad thermal cycler CFX96 Real-Time System (Bio-Rad, Hercules, CA, USA). The amplification programme included enzyme activation at 95°C for 2 min, followed by 40 cycles of 2-step cycling: 95°C for 5 s (denaturation) and 60°C for 10 s (annealing and extension).

Table S1. Primers used for the detection of trophectoderm-specific marker, *GATA3*, and reference genes in bovine blastocysts and immunodissected embryonic cell samples

| **Gene** | **Primer sequence (5’-3’)** | | **Product (bp)** | **NCBI  accession no.** |
| --- | --- | --- | --- | --- |
| *GATA3* | FP | AACATCGACGGTCAAGGCAA | 217 | NM_001076804.1 |
|  | RP | GGTGGATGGACGTCTTGGAG |  |  |
| *YWHAZ* | FP | GATATCTGCAATGATGTACTGTCTCTTTT | 107 | NM_174814.2 |
|  | RP | CGGTAGTAGTCTCCTTTCATTTTCAA |  |  |
| *TBP* | FP | GAATATAATCCCAAGCGTTTTGCT | 103 | NM_001075742.1 |
|  | RP | TGGCTCCTGTGCACACCAT |  |  |
| *H2AFZ* | FP | GCAGGAAATGCATCGAAAGAC | 126 | NM_174809.2 |
|  | RP | AATGACACCACCACCAGCAATT |  |  |
| *B2M* | FP | ATCCAGCGTCCTCCAAAGATTC | 132 | NM_173893 |
|  | RP | CTCCCCATTCTTCAGCAAATCG |  |  |

**Figure S1.** Transcript expression for the trophectoderm (TE) marker *GATA3* was greater (P=0.003) in immunodissected TE than inner-cell mass (ICM) lineages. Low level *GATA3* expression may have arisen as consequence of a few TE cells adhering to the ICM upon separation. Equally, low level *GATA3* expression has been reported in the bovine epiblast and primitive endoderm using different approaches of cell isolation (Ozawa et al., 2012, *BMC Dev Biol* **12**, 33; Negrón-Pérez et al., 2017, *Reprod* **154,** 627), which may be a consequence of low-level residual *GATA3* expression from the morula stage (Saadeldin et al., 2011, *Theriogenology* **75**, 99).

**Supplemental Figure S2.** Follicle size distribution at aspiration differed (χ^2^ = 1231, 4 DF, P<0.001) between stimulated (◼) and non-stimulated (🞏) cycles of OPU.

**Table S2.** Chromosomal errors that can be detected by various means. In this study we employed Karyomapping, Gabriel-Griffin and B Allele Frequency (BAF)/Log R Ratio (LRR) plots

| **Aneuploidy class** | **Detectable by Karyomapping** | **Detectable by Gabriel-Griffin plots** | **Detectable by BAF and LRR** | **Detectable by FISH** | **Detectable by Next Generation Sequencing** |
| --- | --- | --- | --- | --- | --- |
| Trisomy: |  |  |  |  |  |
| Meiotic | Yes | Yes | Yes^‡^ | Yes^‡^ | Yes^‡^ |
| Mitotic | No | No | Yes^‡^ | Yes^‡^ | Yes^‡^ |
| Monosomy: |  |  |  |  |  |
| Meiotic | Yes | Yes | Yes^‡^ | Yes^‡^ | Yes^‡^ |
| Mitotic | Yes* | Yes* | Yes^‡^ | Yes^‡^ | Yes^‡^ |
| Segmental error  (Loss or gain (*de novo*)) | No | No | Yes | No | Yes |
| UPD | Yes | Yes | No | No | No |
| Haploidy | Yes | Yes | Yes | Yes | Yes |
| Triploidy | Yes | Yes | Yes | Yes | Yes |
| Hypotriploidy | Yes | Yes | Yes | No | Yes |
| Mosaicism | No | No | Yes^†^ | Yes | Yes |

*This only works if the monosomy is widespread within the cell population. A mitotic error will most likely cause mosaicism, but a mosaic monosomy will be masked in Karyomapping by the presence of other cells with a correct diploid number. The threshold (i.e. percentage of euploid cells required to mask a mosaic mitotic monosomy) is not known.

^‡^It will detect both but will not distinguish between them

^†^Rough ratios only
